# Deriving causes of child mortality by re–analyzing national verbal autopsy data applying a standardized computer algorithm in Uganda, Rwanda and Ghana

**DOI:** 10.7189/jogh.05.010414

**Published:** 2015-06

**Authors:** Li Liu, Mengying Li, Stirling Cummings, Robert E. Black

**Affiliations:** 1 Department of Population, Family, and Reproductive Health, Johns Hopkins Bloomberg School of Public Health, Baltimore, MD, USA; 2 Institute of International Programs, Johns Hopkins Bloomberg School of Public Health, Baltimore, MD, USA; 3 MEASURE Evaluation, Carolina Population Center, University of North Carolina at Chapel Hill, Chapel Hill, NC, USA; * Joint first authors.

## Abstract

**Background:**

To accelerate progress toward the Millennium Development Goal 4, reliable information on causes of child mortality is critical. With more national verbal autopsy (VA) studies becoming available, how to improve consistency of national VA derived child causes of death should be considered for the purpose of global comparison. We aimed to adapt a standardized computer algorithm to re–analyze national child VA studies conducted in Uganda, Rwanda and Ghana recently, and compare our results with those derived from physician review to explore issues surrounding the application of the standardized algorithm in place of physician review.

**Methods and Findings:**

We adapted the standardized computer algorithm considering the disease profile in Uganda, Rwanda and Ghana. We then derived cause–specific mortality fractions applying the adapted algorithm and compared the results with those ascertained by physician review by examining the individual– and population–level agreement. Our results showed that the leading causes of child mortality in Uganda, Rwanda and Ghana were pneumonia (16.5–21.1%) and malaria (16.8–25.6%) among children below five years and intrapartum–related complications (6.4–10.7%) and preterm birth complications (4.5–6.3%) among neonates. The individual level agreement was poor to substantial across causes (kappa statistics: –0.03 to 0.83), with moderate to substantial agreement observed for injury, congenital malformation, preterm birth complications, malaria and measles. At the population level, despite fairly different cause–specific mortality fractions, the ranking of the leading causes was largely similar.

**Conclusions:**

The standardized computer algorithm produced internally consistent distribution of causes of child mortality. The results were also qualitatively comparable to those based on physician review from the perspective of public health policy. The standardized computer algorithm has the advantage of requiring minimal resources from the health care system and represents a promising way to re–analyze national or sub-national VA studies in place of physician review for the purpose of global comparison.

To accelerate progress toward the Millennium Development Goal 4 (MDG 4) [1] in 2015 by reducing under–five mortality rate by two–thirds since 1990 and end preventable child deaths in a generation [2,3], reliable and updated information on causes of child mortality is critical for prioritizing child health interventions and allocating scarce public health resources. For most low– and middle–income countries (LMICs), distribution of child causes of death is usually derived from community–based verbal autopsy (VA) studies applying systematic modeling [4–6]. In the meantime, an increasing number of national VA studies have been conducted and more are becoming available [6–15]. While empirical data mounts to fill the large information gap of causes of child mortality across LMIC [4,6,16], how to improve consistency and comparability of national VA results should be considered for the purpose of global comparison.

VA studies by design should be consistent and comparable when used to generate population level cause–of–death estimates [17]. In practice, the data collection procedures have been relatively standardized in the national VA studies conducted in the past two decades [7,9–15]. However, when applying national VA data to derive cause–of–death estimates, at least two different methodologies have been applied, including physician review, computer algorithm and probabilistic approaches [17]. The methodological differences in these ascertaining approaches impede direct comparison across estimates.

Compared to computer algorithm, physician review has the apparent disadvantage of involving a large team of physicians. This may intervene with the routine function of the health care systems in many LMICs. In addition, physician review derived cause–of–death estimates may have limited internal consistency due to concerns over repeatability of the approach [7,14,15,17]. Physicians may also interpret VA with subjectivity and judgment [17]. Relying extensively on the open narrative of the circumstances surrounding the death event, physicians can use specific diagnostic techniques differing considerably between individuals and settings. Biased by their prior knowledge of local epidemiology, physicians are also found to be reluctant to assign unexpected causes of death, while favoring some highly specific diagnosis without adequate evidence. Even though two physicians can have high level of agreement, the agreement may simply reflect their similar medical experiences, but does not ensure the results are comparable with those generated in a different time or place [17]. Computer algorithm, in contrast, is considered to be capable of producing more comparable results [17–19].

In this study, we aim to adapt a previously developed standardized computer algorithm [8] to re–analyze national child VA studies conducted recently in Uganda, Rwanda and Ghana to improve the comparability of child cause–of–death information across countries. We also aim to compare our results with those originally derived from physician review to explore issues related with the application of the standardized computer algorithm in place of physician review.

## METHODS

### Ethics statement

The study data were publically accessible and analyzed anonymously. Hence no informed consent was needed.

### National VA studies and the inclusion criteria for re–analysis

The three national Child VA studies re–analyzed includes the 2007 Uganda study, 2008 Rwanda study, and 2008 Ghana study [7,14,15]. The three studies interviewed households with eligible child deaths (plus stillbirths in Uganda) identified through birth history collected in the accompanying Demographic and Health Surveys (DHS) [20–22]. A total of 724, 462 and 226 eligible deaths were identified, among whom 641 (86.4%), 431 (93.3%) and 199 (88.1%) interviews were completed in Uganda, Rwanda and Ghana, respectively. The VA interviews were conducted either following the corresponding DHS, or concurrently with the DHS. **Table 1** provides additional details of the three national VA studies.

**Table 1:** Details of the three national child verbal autopsy (VA) studies

	2007 Uganda Child VA Study	2008 Rwanda Child VA Study	2008 Ghana Child VA Study
**Accompanying Demographic and Health Surveys (DHS)**	Uganda DHS 2006	Rwanda Interim DHS 2007–08	Ghana DHS 2008
**Number of eligible women interviewed in the DHS**	8531	7313	4916
**VA eligibility**	Death of child less than 5 y, or loss of pregnancy of over 6 mo of gestational age, occurring 36 mo preceding the DHS interview	Death of child less than 5 y occurring 36 mo preceding the DHS interview	Death of child less than 5 y occurring after January 2005
**No. of VA eligible deaths**	724	462	226
**No. of completed VA interview**	641	431	199
**VA completion rate (%)**	86.4	93.3	88.1
**Interval between VA initiation and DHS completion (month)**	5	1	0
**Duration of VA interviews**	15 March – 7 April, 2007	20 May – 27 June, 2008	8 September – 25 November, 2008
**Maximum length of recall (month)**	46	42	47

All three VA studies followed the Sample Vital Registration with Verbal Autopsy (SAVVY) protocol [23]. Specifically, the primary caregiver of the deceased child was interviewed about symptoms, signs, and health care received before death with instruments adopted from the standardized WHO VA questionnaires for neonatal and child deaths [24]. Causes of deaths were originally ascertained by two trained physicians independently reviewing both the structured and open–ended narrative sections of the completed VA questionnaires, and coded according to the International Classification of Diseases, 10^th^ revision (ICD–10). If discordant ICD–10 codes were assigned by the two coding physicians, a single cause of death was agreed upon after deliberation.

We did not have access to the open–ended narrative data and focused the current analyses on the structured section of the VA questionnaires. We applied the following criteria to only include children who: 1) were identified as live births; 2) died between ages 0 and 59 months; 3) had complete VA interviews; 4) had sufficient information on age at death; and 5) were administered age–appropriate questionnaire.

### Standardized computer algorithm

**Figure 1:**
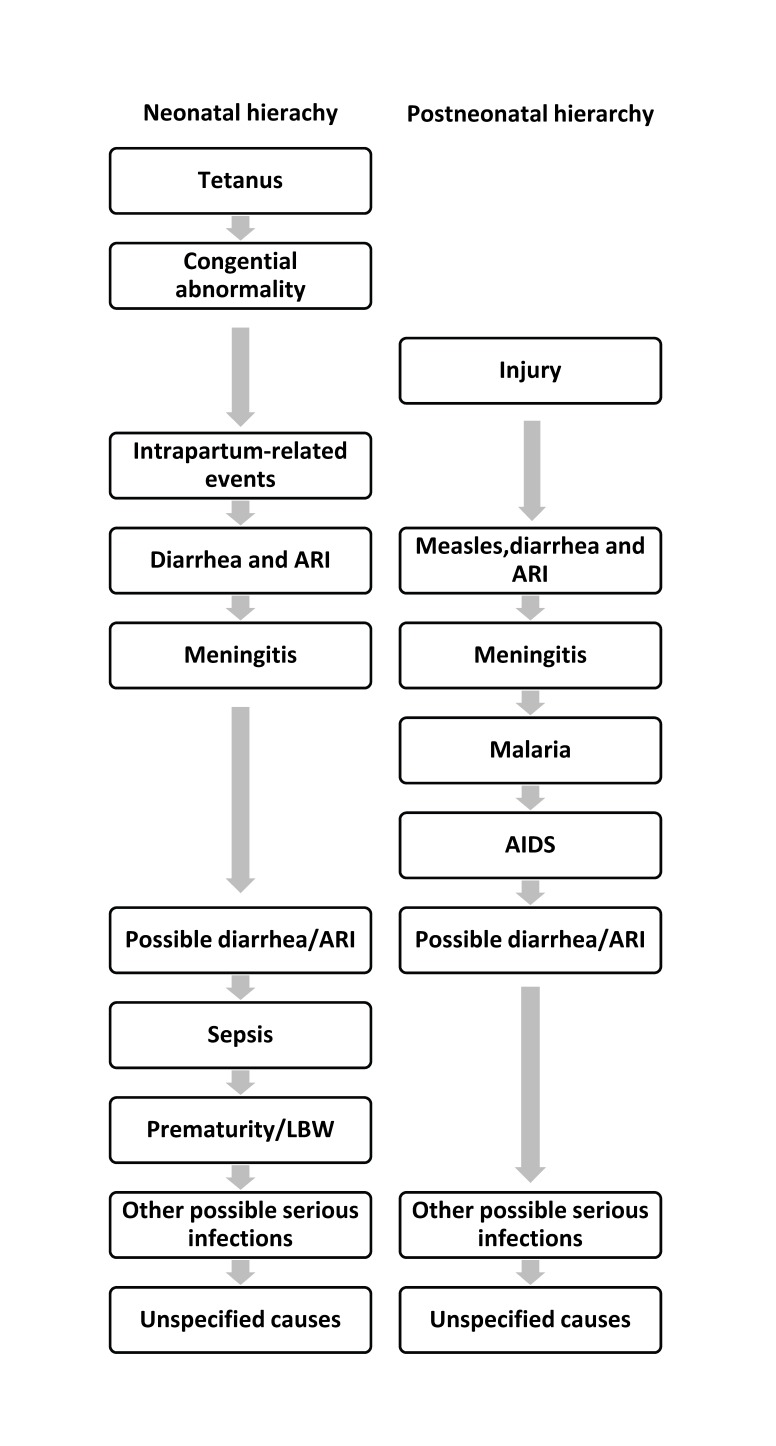
Hierarchy applied in the standardized computer algorithm. This figure provides a visual illustration of the hierarchy used in the algorithm to assign causes of deaths among neonates and children 1–59 months of age.

We adapted a previously developed standardized computer algorithm to assign causes of death [8]. The algorithm was consisted of case definitions and a hierarchical process. The case definitions were combinations of cause–specific signs and symptoms (see **Online Supplementary Document(Online Supplementary Document)**). Generally, the algorithm only allowed one cause for each death. But deaths due to measles, diarrhea and acute respiratory infection (ARI) can be assigned simultaneously, and then re–distributed into the respective single cause according to their cause–specific mortality fractions (CSMFs) assigned before the re–distribution. Causes were assigned through a hierarchical process in which diagnoses with more specific symptoms were made before those with less specific ones, and cases without any diagnosis at the end were classified as unspecified conditions (**Figure 1**). The algorithm contained two parallel hierarchies to assign deaths of children aged 0–27 days and 1–59 months. More information of the standardized computer algorithm can be found elsewhere [8].

Considering the disease profile in Uganda, Rwanda and Ghana, we incorporated the following modifications to the standardized computer algorithm. We added malaria and AIDS to account for the large burden of the two conditions in these countries [6]. Specifically, we adopted the malaria case definition from the WHO standard VA method for infants and children [25], and placed malaria after meningitis/encephalitis in the hierarchy for children aged 1–59 months (**Figure 1**). We chose not to assign deaths due to malaria among neonates due to its low incidence and the associated low accuracy [26].

Due to the lack of information on symptoms of pediatric AIDS cases (eg, oropharyngeal candidiasis, confirmed maternal HIV–1 infection, etc) [27–31], we developed our own preliminary AIDS definition for VA analysis. We first reviewed several pediatric and adult AIDS case validation studies. Then applied major clinical criteria of WHO’s pediatric AIDS case definition in combination with selected available common criteria used in a few adult AIDS case definitions with good validity [28,32–34]. Our final AIDS case definition included any of the following conditions: 1) jaundice; 2) chronic diarrhea lasted for more than 1 month; 3) chronic fever lasted for more than 1 month; 4) wasting, defined as having at least 1 of the following symptoms – paleness, hair color change, edema legs, dry scaly skin; and 5) cough or trouble breathing lasting 3 to 27 days with fever but without a recent diagnosis of tuberculosis. We chose not to assign AIDS among neonates considering the likely low specificity of our preliminary case definition and the low incidence (only approximately 1.6% of all under–five AIDS deaths occur in the first 28 days (personal communication with Neff Walker). Among children aged 1–59 months, AIDS was placed after malaria in the hierarchy.

We also added meningitis/encephalitis and neonatal sepsis in the algorithm. We adopted case definitions of the two conditions from WHO [25], and placed meningitis and neonatal sepsis after ARI and possible diarrhea/ARI, respectively. Additional minor modifications were made to the case definitions to accommodate variations in signs and symptoms collected across countries. A complete list of standardized case definitions applied in this study in comparison with those used previously [8] is provided in **Online Supplementary Document(Online Supplementary Document)**.

### Cause of death categorization and results comparison

We grouped causes of deaths into categories comparable to the Child Health Epidemiology Reference Group (CHERG) categorization, including among neonates: pneumonia, preterm birth complications, intrapartum–related complications (including birth asphyxia and birth injury) [35], sepsis, tetanus, congenital abnormalities, diarrhea and other neonatal disorders; and among children aged 1–59 months: pneumonia, diarrhea, measles, injury, malaria, AIDS, meningitis, and other infections [6]. The group of remaining non–communicable diseases was not assigned separately due to the lack of sufficient information. **Online Supplementary Document(Online Supplementary Document)** maps the ICD–10 codes and the cause categories used in the standardized computer algorithm and physician review.

To compare results derived from standardized computer algorithm vs physician review, individual–level concordance within each country was examined using Cohen’s kappa, where deaths initially assigned to multiple causes (eg, measles, diarrhea and ARI) were excluded. Population level agreement was also assessed by comparing CSMFs and ranking of the CSMFs of the top five single causes [36]. All analyses were conducted using STATA 11 [37] considering the complex survey design.

## RESULTS

Among the 724, 462 and 226 deaths available in the child VA studies in Uganda, Rwanda and Ghana, 530 (126 neonatal and 404 post–neonatal), 360 (121 neonatal and 239 post–neonatal) and 188 (71 neonatal and 117 post–neonatal) deaths met the study inclusion criteria, respectively.

### CSMF derived from the standardized computer algorithm

The CSMFs among neonates, children aged 1–59 months and 0–59 months estimated applying the standardized computer algorithm are presented in **Table 2**. Pneumonia and malaria were the leading causes of deaths across all three countries, contributing around one fifth (16.5–25.6%) of under–five deaths. Diarrhea was responsible for more than one–tenth of under–five deaths in Uganda and Rwanda (15.9% and 12.0%, respectively). Major single neonatal causes in the three countries included intrapartum–related complications (6.4–10.7%) and preterm birth complications (4.5–6.3%). AIDS was also an important cause, contributing 3.0–8.6% of total under–five deaths. Roughly 5% of all under–five deaths were assigned to other conditions across the three countries, and the unspecified causes contributed 8.2–25.0% of total under–five deaths.

**Table 2:** Cause specific mortality fractions among children 0–59 mo ascertained by the standardized computer algorithm, Uganda, Rwanda and Ghana

Age group	Uganda (N = 530)	Rwanda (N = 360)	Ghana (N = 188)
**Neonates aged 0–27 days**			
Pneumonia	2.8	4.4	3.7
Intrapartum–related complications	6.7	10.7	6.4
Preterm birth complications	5.9	4.5	6.3
Congenital abnormalities	1.8	1.9	3.6
Neonatal sepsis	0.4	0.5	0
Other neonatal disorders	2.5	1.5	1.8
Tetanus	2.3	0.9	0.7
Diarrhea	0.2	0.6	0.0
Unspecified	2.1	7.2	13.6
**Children aged 1–59 months**			
Diarrhea	15.7	11.4	2.5
Malaria	23.2	25.6	16.8
AIDS	3.9	3.0	8.6
Injury	2.8	1.2	3.7
Meningitis	1.8	1.2	1.8
Measles	1.6	0.2	0.5
Other infections	2.0	3.3	3.7
Pneumonia	18.3	12.1	14.9
Unspecified	6.1	9.6	11.4
**Total**	**100**	**100**	**100**
**Children aged 0–59 months**			
Pneumonia	21.1	16.5	18.6
Diarrhea	15.9	12.0	2.5
Other conditions	4.5	4.8	5.5
Unspecified	8.2	16.8	25.0

### Agreement between estimates derived from the standardized computer algorithm and physician review

Individual–level concordance between causes assigned by the two approaches is shown in **Table 3**. Only consistent agreement observed in at least two of the three countries are described here in the text. The agreement on injury was at least substantial (kappa ranges 0.61 to 0.80 [38]) in two countries, with the kappa statistics being 0.63 in Uganda and 0.83 in Ghana. The agreement on a number of other causes was at least moderate (kappa ranges 0.41 to 0.60) in two countries, including that on congenital malformation (kappa: 0.57 in Uganda and 0.53 in Ghana) and preterm birth complications (kappa: 0.46 in Uganda and 0.42 in Ghana) among neonates, and malaria (kappa: 0.46 in Uganda and Ghana) and measles (kappa: 0.70 in Uganda and 0.40 in Ghana) among older children. Those causes with fair agreement (kappa ranges 0.21 to 0.40) included intrapartum–related conditions (kappa: 0.30 in Uganda and 0.31 in Ghana) and tetanus (kappa: 0.31 in Rwanda and 0.32 in Ghana) among neonates, and pneumonia (kappa: 0.26 in Uganda and 0.40 in Rwanda), unspecified conditions (kappa: 0.27 in Uganda, 0.25 in Rwanda, and 0.21 in Ghana) and diarrhea (kappa: 0.23 in Uganda and 0.28 in Ghana) among children aged 1–59 months. The rest of the causes all had slight agreement or worse (kappa ranges at or below 0.20) between the two approaches in at least 2 countries.

**Table 3:** Cohen’s kappa (standard error) between results based on computer algorithm and physician review, Uganda, Rwanda and Ghana

Age group	Uganda	Rwanda	Ghana
**Neonates aged 0–27 days:**			
Congenital abnormalities	0.57 (0.039)	0.22 (0.033)	0.53 (0.073)
Preterm birth complications	0.46 (0.042)	0.31 (0.051)	0.42 (0.065)
Intrapartum–related conditions	0.30 (0.040)	0.51 (0.053)	0.31 (0.071)
Pneumonia	0.19 (0.025)	0.25 (0.050)	0.00 (0.000)*
Tetanus	0.16 (0.038)	0.31 (0.050)	0.32 (0.069)
Sepsis	0.09 (0.018)	0.02 (0.032)	0.00 (0.000)*
Other	0.20 (0.035)	0.04 (0.051)	0.14 (0.037)
Unspecified	0.03 (0.042)	0.00 (0.000)*	0.00 (0.000)*
Diarrhea	0.00 (0.000)*	0.00 (0.050)	–†
**Children aged 1–59 months:**			
Injury	0.63 (0.043)	0.44 (0.052)	0.83 (0.073)
Malaria	0.46 (0.043)	0.25 (0.051)	0.46 (0.07)
Measles	0.70 (0.043)	0.00 (0.000)*	0.40 (0.058)
Pneumonia	0.26 (0.041)	0.40 (0.053)	0.16 (0.061)
Unspecified	0.27 (0.043)	0.25 (0.053)	0.21 (0.066)
Diarrhea	0.23 (0.038)	0.19 (0.053)	0.28 (0.07)
Other infections	0.14 (0.032)	0.10 (0.050)	0.28 (0.056)
Meningitis	0.10 (0.035)	0.13 (0.047)	–0.02 (0.071)
AIDS	–0.01 (0.043)	–0.03 (0.046)	0.00 (0.000)*

*0 case is assigned in either the physician review or the algorithm.

†0 case is assigned in both the physician review and the algorithm.

**Figure 2:**
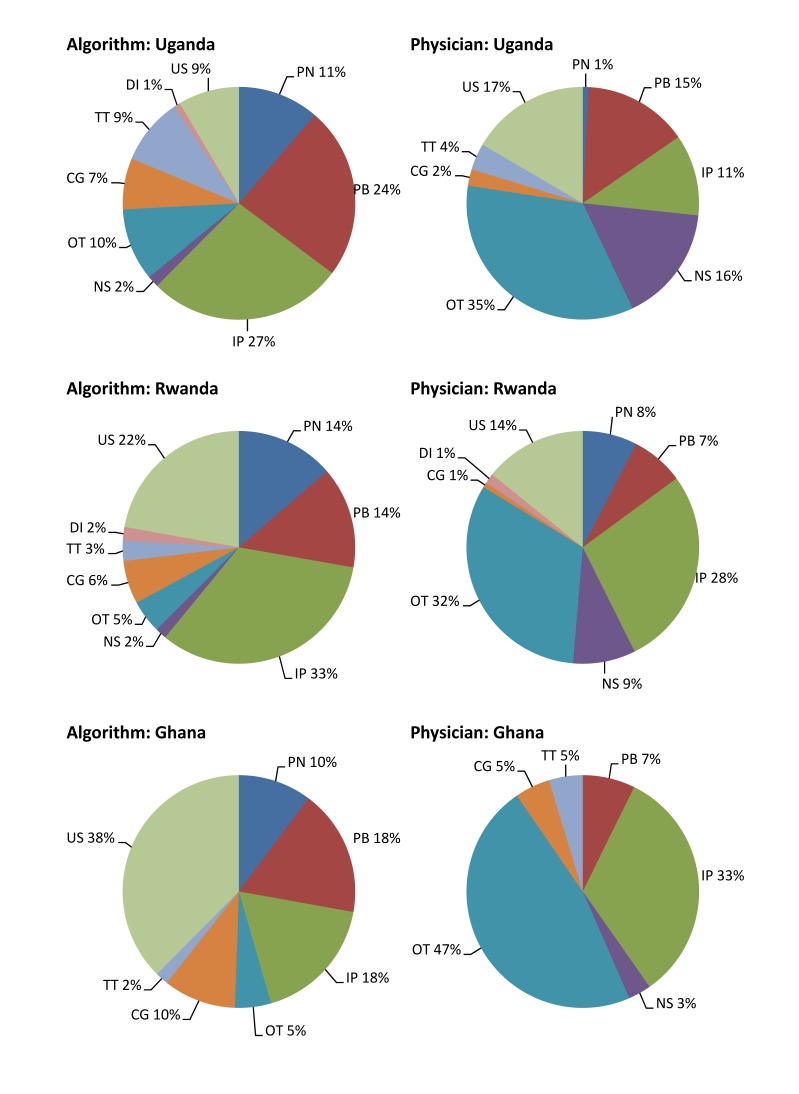
Neonatal cause specific mortality fractions by standardized computer algorithm and physician review, Uganda, Rwanda and Ghana. This figure includes side–by–side pie graphs to compare the cause specific mortality fractions of neonatal deaths generated by standardized computer algorithm and physician review in the three countries. PN – pneumonia; PB – preterm birth/low birth weight; IP – intrapartum–related complications; NS – neonatal sepsis; OT – other neonatal disorders; CG – congenital abnormalities; TT – tetanus; DI – diarrhea; US – unspecified causes.

**Figure 3:**
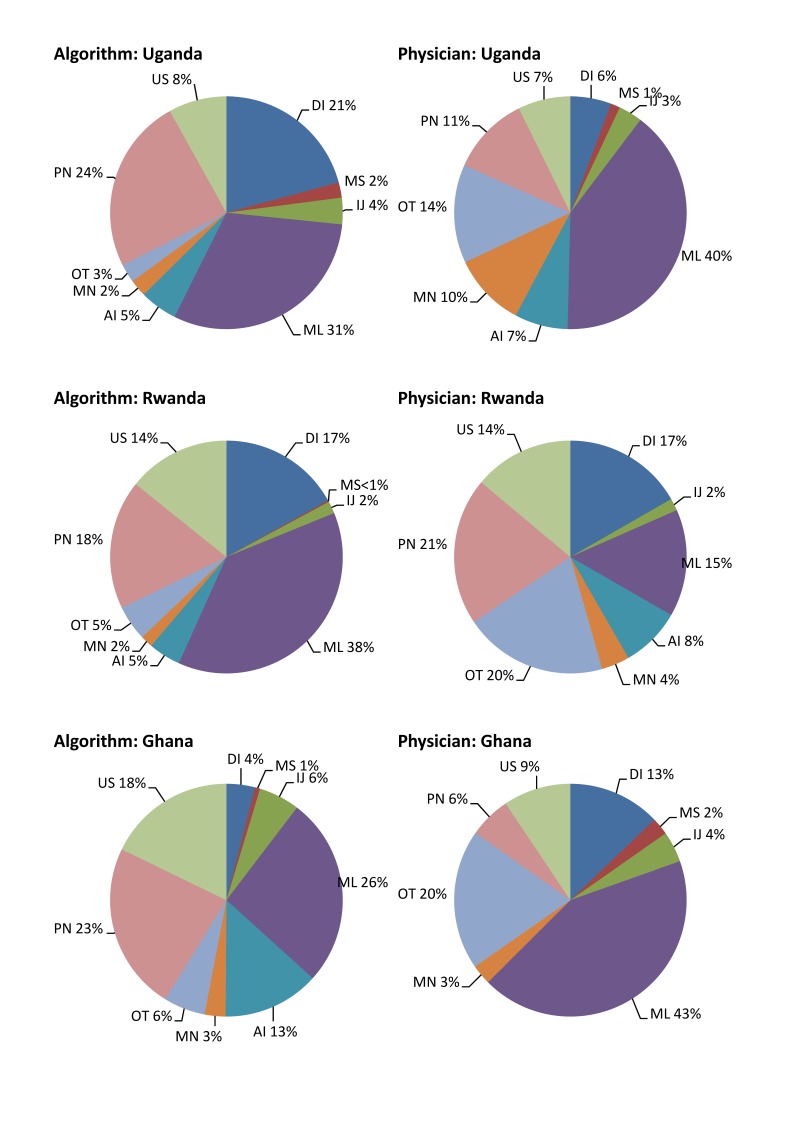
Cause specific mortality fractions among children aged 1–59 months by standardized computer algorithm and physician review, Uganda, Rwanda and Ghana. This figure includes side–by–side pie graphs to compare the cause specific mortality fractions among deaths of children 1–59 months generated by standardized computer algorithm and physician review in the three countries. DI – diarrhea; MS – measles; IJ – injury; ML – malaria; AI – AIDS; MN – meningitis; OT – other infections; PN – pneumonia; US – unspecified causes.

CSMFs derived from the standardized computer algorithm in comparison with those from physician review among neonates and children aged 1–59 months are presented in **Figure 2 and 3**, respectively. The standardized computer algorithm consistently assigned a larger proportion of several causes than physician review among neonates, including pneumonia, preterm births, and congenital abnormalities. Physicians, in contrast, assigned a larger proportion of a few other causes, including neonatal sepsis and other neonatal disorders. Among children aged 1–59 months, no consistent pattern was observed when comparing CSMFs derived from the two methods except that physicians consistently assigned a larger proportion of other infections than the standardized computer algorithm. The discrepancies of malaria–specific mortality fractions derived from the two approaches were huge, ranging between 11 and 23 percentage points, with physicians assigning a higher proportion in Uganda and Ghana and the opposite being true in Rwanda.

The ranking of the top five single causes is show in **Table 4**. Despite discrepancies in the absolute values of CSMFs, the ranking of the top five single causes is similar within each age group across the three countries. Specifically, four out of the five leading causes are the same between results derived by the two approaches. In addition, the ranking is largely similar across countries.

**Table 4:** Ranking of the top five single causes of deaths by age and ascertaining method, Uganda, Rwanda and Ghana

Age group	Rank	Standardized computer algorithm	Physician review
**Uganda**			
**0–27 days**	1	Intrapartum–related complications	Sepsis
	2	Preterm birth complications	Preterm birth complications
	3	Pneumonia	Intrapartum–related complications
	4	Tetanus	Tetanus
	5	Congenital abnormalities	Congenital abnormalities
**1–59 months**	1	Malaria	Malaria
	2	Pneumonia	Pneumonia
	3	Diarrhea	Meningitis
	4	AIDS	AIDS
	5	Injury	Diarrhea
**Rwanda**			
**0–27 days**	1	Intrapartum–related complications	Intrapartum–related complications
	2	Preterm birth complications	Sepsis
	3	Pneumonia	Pneumonia
	4	Congenital abnormalities	Preterm birth complications
	5	Tetanus	Diarrhea
**1–59 months**	1	Malaria	Pneumonia
	2	Pneumonia	Diarrhea
	3	Diarrhea	Malaria
	4	AIDS	AIDS
	5	Meningitis	Meningitis
**Ghana**			
**0–27 days**	1	Intrapartum–related complications	Intrapartum–related complications
	2	Preterm birth complications	Preterm birth complications
	3	Pneumonia	Congenital abnormalities
	4	Congenital abnormalities	Tetanus
	5	Tetanus	Sepsis
**1–59 months**	1	Malaria	Malaria
	2	Pneumonia	Diarrhea
	3	AIDS	Pneumonia
	4	Injury	Injury
	5	Diarrhea	Meningitis

## DISCUSSION

In this paper, we derived comparable causes of child mortality distributions by applying an adapted standardized computer algorithm to re–analyze national VA data collected in Uganda, Rwanda and Ghana in 2007–2008. Overall, the distribution of child mortality is by and large similar across the three countries based on our results, with malaria and pneumonia being the leading causes of under–five deaths and intrapartum–related complications and preterm birth complications being the major neonatal causes.

However, the distribution in Ghana appeared to be different from that in the other two countries in that the diarrhea–specific mortality fraction was much smaller, the AIDS–specific mortality fraction was larger, and more deaths were not assigned. We speculated that the differences between Ghana and the other two countries may be partially attributable to the measurement and data quality issues in the Ghana VA study. Only 2.5% of under–five deaths were assigned to diarrhea in Ghana, which was implausibly low compared to existing studies [6,39]. A closer examination of the Ghana data and our diarrhea case definition revealed that although a large proportion of cases had diarrhea–related symptoms (34% in Ghana, compared to 45% in Uganda and 59% in Rwanda), only a small fraction of these cases had 6 or more stools on days when the symptoms were the most severe (17% in Ghana, compared to 52% in Uganda and 34% in Rwanda). We had no basis to believe that diarrhea symptoms differed between Ghana and the other two countries, and speculated that the discrepancy was partially caused by suboptimal data quality. The suspicion over data quality in Ghana may be further supported by the large AIDS–specific mortality fraction at 8.6%, which was implausibly high given an adult HIV prevalence of 1.8% in 2009 in Ghana [40]. The fact that a quarter of the under–five deaths were not assigned in Ghana, compared to 17% in Rwanda and 8% in Uganda, also raised the concern over quality of the Ghana VA study. One more explanation of the implausibly high AIDS fraction in Ghana could be the result of not assigning sickle–cell disease as a cause of death.

The kappa statistics showed substantial to poor agreement between results generated by the two ascertaining methods, suggesting that the individual–level agreement varied greatly by cause. Causes with more distinct signs and symptoms, such as injury, congenital malformation, preterm birth complications, and measles had a higher level of agreement compared to other causes. Malaria also had moderate agreement, which may be associated with its high prevalence. Other infectious causes like pneumonia, diarrhea, sepsis and AIDS all have fair to poor agreement, probably due to their non–specific symptoms and likely comorbidity with other infectious conditions. It is noted that the low specificity associated with infectious causes is not unique to computer algorithm, rather, it is a common issue shared by all methods ascertaining causes of death [41,42]. Poor agreement among some causes may also have something to do with the fact that we did not have access to the open narrative section of the VA studies. We could have missed useful information on symptoms and signs prior to death that physicians may have had access to [41]. However, the accuracy of causes ascertained at the individual–level is less of a concern as the purpose of the standardized computer algorithm is to derive comparable population–level distribution of child mortality.

The absolute values of CSMFs assigned by the standardized computer algorithm were fairly different from those assigned by physician review among neonates and children aged 1–59 months. However, when ranking of the leading five causes and their specific ranks were compared, many similarities can be drawn between results derived from the two approaches. It suggests that public health policy decisions could be largely similar based on distribution of causes of child mortality derived from both methods [36].

The study has several limitations. First, our preliminary child AIDS case definition has not been validated. The fact that the AIDS–specific fraction did not have a linear relationship with the adult HIV prevalence in the three countries could also suggest that our AIDS case definition have unsatisfactory validity. However, more specific signs and symptoms are generally required to improve the validity of the AIDS case definition. Additional efforts are urgently needed to develop and standardize AIDS case definition among children that can be used in national VA studies.

Second, the standardized computer algorithm assigned 38% of post–neonatal deaths to malaria in Rwanda, which is unlikely to be plausible. CHERG estimated that 3.7% of post–neonatal deaths were attributable to malaria in Rwanda in 2008 [6]. Another independent exercise by WHO applying a natural history model generated an even smaller fraction [43]. If the malaria–specific mortality burden was in fact low in Rwanda, the implausibly high malaria fraction assigned by the standardized computer algorithm was likely caused by the high misclassification error associated with low specificity of the malaria case definition and the low cause–specific fraction [25,44,45]. In fact, given the non–specific symptoms and the associated low specificity [46], concerns have been raised over the suitability of the application of VA to ascertain malaria in low prevalence settings [26,47,48].

In addition, neither the standardized computer algorithm nor physician review is capable of providing the “true” causes of death. Both approaches could be equally invalid. When the two approaches agree, it does not necessarily mean that there is greater truth to the causes assigned.

Despite these caveats, it is feasible to re–analyze national VA studies applying a standardized computer algorithm for the purposes of cross–country comparison and global burden of childhood disease estimation. The standardized computer algorithm produced internally consistent and comparable distribution of causes of child mortality in comparison to physician review. It also has the advantage of requiring minimal resources from the health care system. From the public health policy stand point of view, the standardized computer algorithm and physician review also generate similar sets of leading causes of child deaths. The standardized computer algorithm represents a promising way to re–analyze national or sub–national VA studies in place of physician review. It could be further strengthened with improved validity of child AIDS case definitions. The standardized computer algorithm should be of particular importance in sub–Saharan Africa, where human capital and financial shortfalls are the greatest. The application of a standardized computer algorithm on child VA data are one step forward toward the harmonization of cause–of–death reporting and estimation in children younger than five years. Among the CHERG community, discussion is on–going about how to utilize national VA data so that consistent and comparable cause–of–death estimates can be generated across countries and time for the purpose of global burden of childhood disease estimation. We welcome a discussion on this subject among a wider community.
