# Supplementary material for: Deriving causes of child mortality by re–analyzing national verbal autopsy data applying a standardized computer algorithm in Uganda, Rwanda and Ghana
Source: J Glob Health. 2015 May 19;5(1):010414. doi: 10.7189/jogh.05.010414 (PMC4467513; doi:10.7189/jogh.05.010414)
Supplement: Online Supplementary Document [file jogh-05-010414-s001.pdf]

Online Supplementary Document

Liu and Li et al. Deriving causes of child mortality by re–analyzing national verbal autopsy data applying a standardized computer algorithm in Uganda, Rwanda and Ghana

J Glob Health 2015;5:010414

Appendix 1: Case definitions used in this study in comparison with the case definitions used in Liu et al

Cause of deaths	Case definition used in this study	Case definitions used in Liu et al
**Neonatal tetanus**	a. Age at death: 4-14 days;<br>AND<br>b1. Convulsion;<br>OR<br>b2. Muscle spasms;<br>AND<br>c. Suckled normally after birth but stopped suckling.	a. Age at death: 4-14 days;<br>AND<br>b. Convulsions;<br>AND<br>c1. Cried normally after birth but stopped crying in the final illness;<br>OR<br>c2. Suckled normally after birth but stopped suckling in the final illness.
**Congenital abnormality**	a. Age at deaths<=28 days;<br>AND<br>b1. The child have any malformation at birth;<br>OR<br>b2. The child had a reported malformation at birth.	a. Age at death<=28 days;<br>AND<br>b1. There was something physically wrong with the baby at birth;<br>OR<br>b2. The child had a reported malformation at birth.
**Injury**	a. Age at deaths>=29 days;<br>AND<br>b. Accidental or injury deaths including drowning.	a. Age at death: >=29 days;<br>AND<br>b. Accidental or injury deaths including drowning.
**Birth asphyxia**	a. Age at death <=7 days;<br>AND<br>b. Not able to cry normally after birth;<br>AND<br>c1. Not able to breathe after birth;	a. Age at death: <=7 days;<br>AND<br>b. Not able to cry normally after birth;<br>AND<br>c1. Not able to breathe after birth;

**Cause of deaths**	**Case definition used in this study**	**Case definitions used in Liu et al**
	OR<br>c2. Not able to suckle normally after birth.	OR<br>c2. Not able to suckle normally after birth.
**Birth injury**	a. Age at death <=7days;<br>AND<br>b. Bruises or marks of injury on the body or head at birth.	a. Age at death: <=7 days;<br>AND<br>b. Bruises or marks of injury on the body or head.
**Measles**	a. Age at death>=6 months;<br>AND<br>b. Measles-type rash on body and face;<br>AND<br>c. Accompanied by fever;<br>AND<br>d. With at least 1 of the following specific symptoms: dry cough, or red or runny eyes.	a. Age at death: >=6 months;<br>AND<br>b. Measles-type rash without water in the eruptions on body and fact that appeared in 3 months before death;<br>AND<br>c. Accompanied by fever;<br>AND<br>d. With at least 1 of the following specific symptoms: dry cough, red or runny eyes, or running nose.
**Meningitis/encephalitis**	a. Age at death>=29 days;<br>AND<br>b. Fever;<br>AND<br>c. Convulsions;<br>AND<br>d1. Stiff neck;<br>OR<br>d2. Bulging fontanelle;<br>AND<br>e. With at least 1 of the following specific symptoms: unconscious.	N/A
**Malaria**	a. Age at death>=29 days;<br>AND<br>b. Fever;<br>AND<br>c. With at least 1 of the following specific symptoms: convulsions, difficult breathing, unresponsive, or pallor;	N/A

Cause of deaths	Case definition used in this study	Case definitions used in Liu et al
	AND<br>d. No stiff neck or bulging fontanelle.	
**AIDS**	a. Age at death>=29 days;<br>AND<br>b1. Jaundice;<br>OR<br>b2. Chronic diarrhea > 1 month;<br>OR<br>b3. Chronic fever > 1 month;<br>OR<br>b4. Wasting defined as having all the 4 following symptoms: paleness, hair color change, edema legs, dry scaly skin;<br>OR<br>b5. Cough or trouble breathing lasting 3-27 days with fever but not recent TB.	N/A
**Diarrhea**	**Neonatal diarrhea**<br>a. Age at death<=28 days;<br>AND<br>b. Diarrhea was reported with a peak of 6 or more stools in 24 hours.<br><br>**Postneontal diarrhea**<br>a. Age at death>=29 days;<br>AND<br>b1. Frequent loose or liquid stools starting from 1 to 13 days before death and continued until death;<br>AND<br>b2. With a peak number of 6 or more stools in 24 hours;<br>AND<br>b3. At least 2 of the 6 following specific symptoms were reported: weakness, dry mouth, sunken eyes, loose skin,	**Neonatal diarrhea**<br>a. Age at death<=28 days;<br>AND<br>b. Frequent liquid or watery or loose or soft stools or diarrhea was reported with a peak of 6 or more stools in 24 hours.<br><br>**Postneontal diarrhea**<br>a. Age at death>=29 days;<br>AND<br>b1. Frequent loose or liquid stools starting from 1 to 13 days before death and continued until death;<br>AND<br>b2. With a peak number of 6 or more stools in 24 hours;<br>AND<br>b3. At least 2 of the 6 following specific symptoms were reported: weakness, dry mouth, sunken eyes, loose skin,

Cause of deaths	Case definition used in this study	Case definitions used in Liu et al
	depressed fontanels, and no or very little urine;<br>OR<br>c. Frequent loose or liquid stools started from at least 14 days prior to death and continued until death.	depressed fontanels, and no or very little urine;<br>OR<br>c. Frequent loose or liquid stools started from at least 14 days prior to death and continued until death.
**Acute Respiratory Infection (ARI)**	**Neonatal ARI**<br>a. Age at death<=28 days ;<br>AND<br>b. difficult breathing started at least 1d before death and;<br>AND<br>c. Had at least 2 of the 3 specific symptoms: grunting, nostril flaring, and chest indrawing.<br><br>**Postneonatal ARI**<br>a. Age at death>=29 days;<br>AND<br>b1. Had a cough that started at least 3 days before death;<br>OR<br>b2. Difficulty breathing that started at least 1 day before death;<br>AND<br>c. Had at least 2 of the following 6 specific symptoms: noisy breathing, grunting, wheezing, nostril flaring, or chest indrawing.	**Neonatal ARI**<br>a. Age at death<=28 days;<br>AND<br>b. Difficult breathing started at least 1 day before death and lasted until death;<br>AND<br>c. Had at least 2 of the following 3 specific symptoms: grunting, nostril flaring, and chest indrawing.<br><br>**Postneonatal ARI**<br>a. Age at death>=29 days;<br>AND<br>b1. Had a cough that started at least 3 days before death and lasted at least until the day before death;<br>OR<br>b2. Difficult breathing that started at least 1 day before death and lasted until death;<br>AND<br>c. Had at least 2 of the following 6 specific symptoms: noisy breathing, stridor, grunting, wheezing, nostril flaring, or chest indrawing.
**Possible pneumonia**	**Neonatal possible pneumonia:**<br>a. Age at death<=28 days ;<br>AND<br>b. Had at least 2 of the following signs of serious infection: stopped suckling, difficult breathing, chest indrawing, convulsions, and fever;<br>AND	**Neonatal possible pneumonia**<br>a. Age at death<=28 days;<br>AND<br>b. Had at least 2 of the following signs of serious infection: stopped suckling, difficult breathing, chest indrawing, convulsions, and fever;<br>AND

Cause of deaths	Case definition used in this study	Case definitions used in Liu et al
	c. Difficult breathing.<br><br>**Postneonatal possible pneumonia**<br>a. Age at death>=29 days;<br>AND<br>b. Had at least 2 of the following signs of serious infection: difficult breathing, chest indrawing, convulsions, and fever;<br>AND<br>c. Cough or difficult breathing.	c. Difficult breathing.<br><br>**Postneonatal possible pneumonia**<br>a. Age at death>=29 days;<br>AND<br>b. Had at least 2 of the following signs of serious infection: difficult breathing, chest indrawing, convulsions, and fever;<br>AND<br>c. Cough or difficult breathing.
**Possible diarrhea**	**Neonatal possible pneumonia:**<br>a. Age at death<=28 days ;<br>AND<br>b. Had at least 2 of the following signs of serious infection: stopped suckling, difficult breathing, chest indrawing, convulsions, and fever;<br>AND<br>c. Diarrhea.<br><br>**Postneonatal possible pneumonia:**<br>a. Age at death>=29 days ;<br>AND<br>b. The child had 2+ signs of serious infection: difficult breathing, chest indrawing, convulsions, and fever;<br>AND<br>c. Diarrhea.	**Neonatal possible diarrhea**<br>a. Age at death<=28 days;<br>AND<br>b. Had at least 2 of the following signs of serious infection: stopped suckling, difficult breathing, chest indrawing, convulsions, and fever;<br>AND<br>c. Loose or liquid stools.<br><br>**Postneonatal possible diarrhea**<br>a. Age at death>=29 days;<br>AND<br>b. Had at least 2 of the following signs of serious infection: difficult breathing, chest indrawing, convulsions, and fever;<br>AND<br>c. Loose or liquid stools.
**Prematurity/ Low Birth Weight**	a. Age at death<=28 days ;<br>AND<br>b1. Pregnancy ended early (<=7 months);<br>OR<br>b2. the baby was reported to be very small or smaller than usual.	a. Age at death<=28 days;<br>AND<br>b1. Pregnancy ended early (<=7 months);<br>OR<br>b2. The baby was reported to be very small or smaller than usual.
**Other**	**Neonatal other possible serious infections**	**Neonatal other possible serious infections**

Cause of deaths	Case definition used in this study	Case definitions used in Liu et al
**possible serious infections**	a. Age at death<=28 days;<br>AND<br>b. Had at least 2 of the following signs of serious infection: stopped suckling, difficult breathing, chest indrawing, convulsions, and fever.<br><br>**Postneonatal possible serious infections**<br>a. Age at death>=29 days ;<br>AND<br>b. Had at least 2 of the following signs of serious infection: difficult breathing, chest indrawing, convulsions, and fever.	a. Age at death<=28 days;<br>AND<br>b. Had at least 2 of the following signs of serious infection: stopped suckling, difficult breathing, chest indrawing, convulsions, and fever.<br><br>**Postneonatal other possible serious infections**<br>a. Age at death>=29 days;<br>AND<br>b. Had at least 2 of the following signs of serious infection: difficult breathing, chest indrawing, convulsions, and fever.
**Unspecified causes**	The remaining deaths.	The remaining deaths.

Appendix 2. The final cause of death categories, causes of deaths assigned in the standardized computer algorithm, and their corresponding ICD-10 codes

Final cause of death categories used in the standardized algorithm and physician review	Intermediate cause of deaths assigned in the standardized computer algorithm	ICD-10 code
	**Neonates aged 0-28 days**	
Pneumonia	pneumonia, possible pneumonia, (a proportion of) pneumonia and diarrhea, (a proportion of) possible pneumonia and diarrhea	H65-H66, J00-J22, J85, P23
Preterm/LBW	prematurity, low birth weight	P01.0, P01.1, P07, P22, P25-P28, P61.2, P77
Intrapartum-related events	birth asphyxia, birth injury	P01.7-P02.1, P02.4-P02.6, P03, P10-P15, P20-P21, P24, P50, P90-P91
Sepsis	sepsis	A34, A35, A40-A41, P36
Tetanus	tetanus	A33
Other conditions	meningitis, possible other infection	R00-R99, P95, all the cases without a specific diagnosis
Congenital	congenital abnormality	Q00-Q99 (all Q)
Diarrhea	diarrhea, possible diarrhea, (a proportion of) pneumonia and diarrhea, (a proportion of) possible pneumonia and diarrhea	A00-A09
Unspecified	unspecified causes	R00-R99, P95, all cases without a diagnosis
	**Children aged 1-59 months**	
Diarrhea	diarrhea, possible diarrhea, (a proportion of) pneumonia and diarrhea, (a proportion of) possible pneumonia and diarrhea	A00-A09
Measles	measles only, measles followed by pneumonia or diarrhea	B05
Injury	injury	V01-Y89 (all v, w, x and y except y90-y98)
Malaria	malaria	B50-B54
AIDS	AIDS	B20-B24
Meningitis	meningitis	A39, A83, A84, A87, G00, G03, G04
Other communicable diseases	malnutrition, other possible serious infections	All the cases with a specific diagnosis (excluding R00-R99, P95)
Pneumonia	pneumonia, possible pneumonia, (a proportion of) pneumonia and diarrhea, (a proportion of) possible pneumonia and diarrhea	H65-H66, J00-J22, J85, P23
Unspecified	unspecified causes	R00-R99, all cases without a diagnosis
